# How Duty-Free Policy Influences Travel Intention: Mediating Role of Perceived Value and Moderating Roles of COVID-19 Severity and Counterfactual Thinking

**DOI:** 10.3389/fpsyg.2022.908736

**Published:** 2022-06-17

**Authors:** Yajun Xu, Wenbin Ma, Xiaobing Xu, Yibo Xie

**Affiliations:** Management School, Hainan University, Haikou, China

**Keywords:** duty-free policy, perceived value, COVID-19 severity, counterfactual thinking, travel intention

## Abstract

Counterfactual thinking is presumed to play a preparatory function in promoting people’s behavioural intentions. This study specifically addresses the impacts of COVID-19 severity, tourists’ counterfactual thinking about the pandemic, and tourists’ perceived duty-free consumption value on the effect of a duty-free policy on travel intentions. Four hundred and ten participants took part in this study, which involved a 2 (duty-free policy: absent vs. present) × 2 (COVID-19 severity: high vs. low) design. Results reveal the following patterns: (a) compared to the absence of a duty-free policy in tourist destinations, enactment of a duty-free policy leads to stronger visit intentions through greater perceived value and (b) the effect of a duty-free policy on travel intention is moderated by tourists’ counterfactual thinking and COVID-19 severity.

## Introduction

Global tourism has expanded rapidly in recent years, framing this industry as a growth point and an important aspect of various countries’ economies (Richards, 2011). Duty-free shopping in particular has ballooned in tourist destinations and led to several benefits: wider employment, heightened consumption, and accelerated economic development (Sohn and Lee, 2017). Because duty-free shopping policies can promote the tourism economy’s development and improve national welfare, many destinations have begun to attract tourists through such policies (Martín et al., 2019).

However, the tourism industry is inherently sensitive and subject to uncertainties (Sharma, 2013). The coronavirus disease 2019 (COVID-19) pandemic has brought incalculable losses to this economic sector that relies heavily on human mobility (Tong et al., 2021). Specifically, the COVID-19 pandemic has resulted in the loss of one billion tourists worldwide, causing the loss of 180 million jobs (56.7% of the total world travel and tourism contribution to employment) (Škare et al., 2021). Resultant pressure suggests that governments should leverage tools such as duty-free policies to stimulate tourism development. It is accordingly crucial to study the mechanisms behind the effects of these policies on tourists’ travel intentions amid the pandemic. Specifically, more remains to be discovered about how tourists’ psychological reactions to the pandemic shape the impacts of duty-free policies on tourists’ willingness to travel. This article explores whether a duty-free policy has a greater or lesser effect on tourists’ travel intentions if they exhibit counterfactual thinking (i.e., imagining how things might have been different in the past), such as “I could have enjoyed my trip if there had not been a COVID-19 pandemic.”

The literature on tourist destinations’ duty-free policies has largely focused on qualitative analyses of policy implementation and associated shortcomings, demonstrating the role of duty-free policies in promoting travel intentions (e.g., Dimanche, 2003; Lin and Chen, 2013; Kim et al., 2019; Martín et al., 2019). However, there has been less discussion of the specific mediating mechanisms (e.g., consumer perceived value) through which duty-free policies affecting travel intentions, and there is a lack of behavioral theory (e.g., functional theory of counterfactual thinking) to explore the moderating variables.

The present paper is innovative in its empirical approach to investigating the effect of duty-free policies on tourists’ destination visit intentions amid the pandemic. Specifically, we propose that counterfactual thinking plays a preparatory function in promoting people’s behavioural intentions through the functional theory of counterfactual thinking (Epstude and Roese, 2008; Huang et al., 2021). More precisely, how tourists’ behavioural intentions toward a destination are generated and which factors influence such intentions are of interest to destination marketers: behavioural intentions (e.g., travel intentions) can forecast tourists’ travel behaviour. In essence, this paper considers the roles of COVID-19 severity, tourists’ counterfactual thinking about the pandemic, and tourists’ perceived duty-free consumption value on the impact of a duty-free policy on travel intentions. Results offer theoretical and practical insight related to duty-free policies in tourist destinations during the pandemic.

## Literature Review

### Duty-Free Policies

Research has shown that tourism is well suited to taxation given the industry’s inherent diversity, mobility, and dependence on public resources (Gooroochurn and Sinclair, 2005; Cooper and Hall, 2008; Durán Román et al., 2020). Tourism taxation is notably relevant to governments, businesses, and tourists (Christie and Morrison, 1985; Gunn and Var, 2002). However, tourism taxation can have negative consequences (Mak, 1988; Jensen and Wanhill, 2002): excessive taxes may inhibit tourism demand and weaken destinations’ competitiveness, heavily burdening both businesses and tourists (Hall, 2000; Ryan, 2002; Palmer and Riera, 2003).

Meanwhile, many destinations have started to implement duty-free policies to stimulate tourism development (Martín et al., 2019). A duty-free policy is a tax incentive intended to promote departing tourists’ duty-free shopping. Tourists can purchase from duty-free stores or approved online sales portals and retrieve their products at designated areas in airports, railway stations, or ports when leaving (Camilleri, 2018). This type of policy reduces customs duty taxes, valued added tax, and consumption tax (Bouët et al., 2012). Duty-free policies also represent a typical tax policy to spark tourism consumption, meant to encourage tourists to shop in destinations and to consume products (Kim et al., 2019). Studies have shown that a duty-free policy can enhance individuals’ travel intentions, tourism spending, and tourism revenue. For example, Facchini and Willmann (1999) examined the welfare benefits of duty-free shopping and discovered that a duty-free policy produced higher Pareto efficiency than a free trade policy. Christiansen and Smith (2001) observed that a duty-free policy contributed to socioeconomic welfare through positive effects on tourist spending, commodity pricing, and tourism revenue.

As another example, the enactment of a duty-free policy in Hainan, China has attracted empirical attention. Liu et al. (2015) performed multiplicative interaction analysis to compare second-hand data from China’s Hainan and Guangxi provinces. The authors concluded that a duty-free policy could effectively foster economic growth in Hainan province. Other scholars have pointed out that, as individuals’ disposable income increases, a duty-free policy can boost domestic tourism income (Qi et al., 2013). Luo and Tian (2016) studied the effects of a duty-free policy and found that raising duty-free limits, improving the shopping environment, and introducing more duty-free brands could significantly increase sales of duty-free products. Dimanche (2003) assessed international tourists’ shopping behaviour during 10 years of duty-free policy implementation in the US state of Louisiana. Findings indicated that the policy incentivised tourists to purchase more products, while adjusted tax rates on duty-free products played a major role in the policy’s impact.

### Counterfactual Thinking

Counterfactual thinking is a thinking process in which individuals substitute unreal conditions or possibilities (Kahneman and Tversky, 1981). People often contemplate how things could have been different under other circumstances. For example, when people reflect on their lives, they sometimes realise how many of their dreams have gone unfulfilled and how many opportunities have passed them by. They may reflect about negative outcomes, thinking these may not have happened if only one or more aspects of the past had been different. This process of mentally generating better alternatives to a factual state of affairs is called upward counterfactual thinking (Roese, 1997; Epstude and Roese, 2008; Byrne, 2016; Broomhall et al., 2017). Its key feature is the juxtaposition of one’s current status against an imagined better alternative state (Markman et al., 1993; Markman and McMullen, 2003; Epstude and Roese, 2008).

Specifically, consider a traveller who rushes to the train station only to find that their train left 5 minutes ago: they may think, “If I hadn’t gotten caught in that traffic jam, I would have arrived at the train station on time.” Similarly, when faced with the impact of the COVID-19 pandemic, a person may think, “I would have been able to travel and enjoy my trip if there were no pandemic.” Counterfactual thinking is a mental simulation of alternatives to one’s reality (Kahneman and Tversky, 1982; Roese, 1997). In this form of thinking, one compares actual outcomes with those that could have occurred (Roese and Olson, 2014). Counterfactual thinking is closely tied to emotions such as regret, guilt, and shame (Mandel and Dhami, 2005; Byrne, 2016). It is also functional in that it can guide future behaviour (Roese and Olson, 1997). Through counterfactual thinking, people can justify failed attempts to explain the past and prepare for the future; the resulting behavioural intention helps to ensure better outcomes (Byrne, 2016).

The functional theory of counterfactual thinking, as proposed by Epstude and Roese (2008), focuses on how such thinking affects subsequent behaviour. Counterfactual thinking thus represents a useful and necessary part of behavioural regulation. When a situation does not align with one’s ideal state, the person generally adapts their behaviour to achieve equilibrium (Russell, 2003). Behavioural regulation usually begins with a problem or other negative experience that defies expectations and in turn counterfactual thinking. Such thinking typically extrapolates the antecedents that would inspire a person to pursue change and different behaviour, hence the functional theory. Scholars (e.g., Krishnamurthy and Sivaraman, 2002; Page and Colby, 2003) have demonstrated that counterfactual thinking can facilitate behavioural intention.

### COVID-19 Severity

Coronavirus disease 2019 refers to pneumonia caused by a novel coronavirus infection (Velavan and Meyer, 2020). At present, the pandemic continues to worsen, with recurrent and relatively uncontrolled outbreaks in many countries (Shi et al., 2020). This pandemic has intensified anxiety among the general public; for the tourism industry, COVID-19 has also greatly diminished individuals’ willingness to travel (Fotiadis et al., 2021). As of April 2022, the COVID-19 has infected more than 500 million people and caused more than six million deaths (Johns Hopkins University COVID-19 Dashboard). In addition, the COVID-19 pandemic can affect the behaviour of tourists. For example, many tourists cancel their planned trips due to fear of risk because it is difficult to avoid COVID-19 infection during the trip (Abbas et al., 2021).

The ripple effect (e.g., Kasperson et al., 1988) can partly explain the impact of COVID-19 severity. This effect suggests that the closer one is to the centre of a crisis event, the higher one’s risk perceptions and negative emotions about that event (Slovic, 1987; Kasperson et al., 1988; Barsade, 2002). “Ripple” is a metaphor depicting a risk event’s impact within a risk amplification framework (Kasperson et al., 1988), such as a stone thrown into a calm lake (i.e., the point of contact is most volatile, and the surrounding water gradually becomes less volatile as the distance from the point of contact increases). Research has further shown that, when defining COVID-19 severity by geographic distance, residents closer to the pandemic centre exhibit stronger risk perceptions and anxiety than those in more distant areas (Wen et al., 2020).

### Consumers’ Perceived Value

Consumers’ perceived value embodies the difference between the perceived worth of a good and its actual price (Sweeney and Soutar, 2001; Roberts et al., 2017). After perceiving the value of a product or service, a person’s subjective evaluation of its value minus the cost paid represents the consumer perception effect (Yu and Lee, 2019). Consumers’ *perceived* value differs from a product’s or service’s *objective* value (Morar, 2013). Perceived value is essentially the trade-off between perceived value and perceived costs (Sánchez-Fernández and Iniesta-Bonillo, 2007). Perceived value is also personalised; that is, consumers do not perceive the same product or service as having the same value. Further, due to the trade-off between value and cost, individuals’ consumption decisions are based on perceived value rather than a single factor (Loureiro et al., 2012).

The money and other resources (e.g., time, energy, effort) that consumers expend to obtain a product or service affect perceived value (Chi and Kilduff, 2011). Reducing monetary expenditure increases perceived value for consumers with high price sensitivity; reducing time and energy expenditure is more noteworthy for those with low price sensitivity (Chua et al., 2015). Few consumers seriously consider price and benefits when assessing a product’s value, instead relying on external cues to form value-related impressions (Beverland et al., 2008). Consumers therefore make purchases upon processing only a small amount of acquired information (Dodd et al., 2005). Perceived value depends on the reference system through which a consumer perceives value (i.e., the context in which the valuation takes place) (Wu et al., 2014). For instance, consumers perceive value distinctly at specific consumption locations and times.

### Travel Intention

Travel intention refers to tourists’ preferences to visit a destination based on relevant knowledge (Jang et al., 2009). Travel intention is mostly influenced by external stimuli, personal needs and desires, external factors, and destination characteristics. External stimuli spur one’s desire to travel, for example, marketing campaigns, the promotion of destinations et al. (Reza Jalilvand et al., 2012). Needs and desires are a set of attributes such as personal attitudes (Mohsin et al., 2017), values, and traits (Tepavčević et al., 2021). External factors include destination image, past travel experience, time, and economic variables (Hsieh et al., 2016; Rasoolimanesh et al., 2021; Yao et al., 2021). Destination characteristics consist of elements such as destination services. The factors influencing travel intention can be categorised into push and pull motivations, in which push motivations are derived from tourists’ desire and need to travel, whereas pull motivations arise from a destination’s appeal to the tourist, stimulating tourists’ interest in it (Klenosky, 2002).

Many researchers have also explored travel intentions in the context of COVID-19 pandemic. For example, Tepavčević et al. (2021) found that travel intention and fear of COVID-19 pandemic are influenced by different personality traits. In addition, different fears of COVID-19 (infection during travel, as well as lack of funds, and loss of job during the critical period of COVID-19 pandemic) reduce travel intention (Gajić et al., 2021). Besides, researchers have found that tourists’ perceptions of COVID-19 severity had a significant and negative impact on willingness to travel by public transportation (Liu et al., 2022). This suggests that tourists tend to use safer (in terms of infection) modes of travel during the COVID-19 pandemic (De Vos, 2020). Also, during the COVID-19 pandemic, social media plays an important role for travel intention through destination image restoration and reputation repair, allowing tourist destinations to use digital platforms to enhance the brand image of the destination and enhance the travel intentions of tourists (Rastegar et al., 2021). It has also been shown that more distant destinations are associated with more uncertainty in the COVID-19 pandemic, and are perceived to be riskier than nearby destinations. As a result, tourists show higher travel intention for short-haul travel plans than for long-haul travel plans (Williams et al., 2022).

### Theoretical Basis and Research Model

Our proposed research model is grounded in perceived value theory. Zeithaml (1988) considered perceived value through the lens of consumer psychology, describing it as the perceived overall value of a product/service following a consumer’s thorough assessment of associated costs and benefits. Best (2013) classified perceived value based on emotional benefits, economic benefits, and perceived benefits. Burns (1994) summarised perceived value across four dimensions—use value, product value, evaluation value, and possession value—and asserted that because value is consumer-specific, marketers should assume a consumer perspective. Put simply, consumers’ perceived value moulds purchase behaviour.

Sheth et al. (1991) developed a broader theoretical framework of perceived value and distinguished the concept using five dimensions (i.e., functional value, social value, emotional value, satisfaction value, and conditional value). They stated that consumption decisions result from these five variables and that the effect of each on consumption choices differs contextually. Lai (1995) regarded consumers’ perceived value from a product perspective, a social perspective, and a consumer perspective. The product view pertains to a product’s functional value; the social view entails social value and ecological value; and the consumer view concerns experiential value, emotional value, aesthetic value, satisfaction value, and situational value. According to value model, perceived value is based on consumer preferences for a product and varies across consumers for the same product (Peloza and Shang, 2011). Depending on who benefits, consumers’ perceived value is divided into self-directed value (where the consumer personally benefits) and social-directed value (where others benefit). When a product provides social-directed value, consumers perceive that self-directed value is impaired. When a product provides social-directed value, consumers perceive that self-directed value is impaired (Obermiller et al., 2009).

**FIGURE 1:**
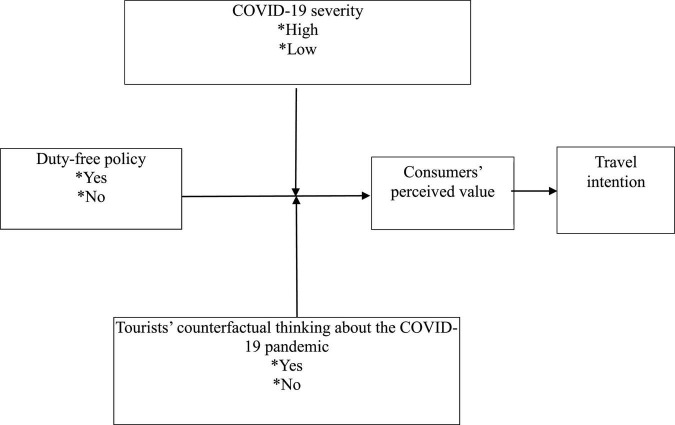
Proposed research model.

In the tourism field, perceived value theory initially focussed on the hotel industry (e.g., Bojanic, 1996). The theory has since expanded to domains such as leisure and entertainment (Petrick et al., 2001), travel agencies (Sánchez et al., 2006), and restaurants (Hyun et al., 2011). We developed our research model based on existing theory, taking a duty-free policy as the independent variable, COVID-19 severity and COVID-19 counterfactual thinking as moderators, consumers’ perceived value as the mediator, and travel intention as the dependent variable (Figure 1).

## Hypothesis Development

### Consumers’ Perceived Value Mediates the Effect of a Duty-Free Policy on Travel Intention

Research has highlighted the positive impact of perceived value on behavioural intention. For example, when studying the outlet market for Australian coffee, Chen and Hu (2010) discovered that perceived value significantly and positively influenced consumers’ loyalty. Higher perceived value can shape consumers’ future behavioural intentions in two ways: by informing product preferences and by encouraging word-of-mouth communication about the product. Wang and Wang (2010) examined users of hotel reservation systems and found perceived value to indirectly affect behavioural intention through the mediating role of satisfaction. In a cruise context, Duman and Mattila (2005) observed that perceived value shaped tourists’ behavioural intentions more strongly than satisfaction. Gallarza and Gil Saura (2006) further found that perceived value promoted tourists’ loyalty.

In the tourism domain, Petrick (2004) discerned that consumers’ perceived value of tourism products through travel experiences positively contributed to recommendation intentions. Others have suggested that the higher the perceived value, the stronger one’s intentions to purchase and recommend a tourism product (Duman, 2002; Kleijnen et al., 2007). We therefore presume that the higher a destination’s perceived value, the stronger tourists’ intentions to visit. More precisely:

H1: Compared to the absence of a duty-free policy in a tourist destination, implementation of a duty-free policy will generate stronger travel intention through greater perceived value.

### Moderating Role of COVID-19 Severity

The COVID-19 pandemic shares characteristics with other crisis events (e.g., earthquakes) such as abruptness, urgency, ambiguity, and widespread social impact (Jia et al., 2020). However, COVID-19 is highly infectious and has a long incubation period, amplifying its uncontrollability and posing a demonstrable threat to people’s lives and health (Fang et al., 2020). The pandemic has thus elicited intense public fear (Zuo et al., 2020). From a self-protection standpoint, people in different areas face varying degrees of COVID-19 severity: residents in the epicentre of a COVID-19 outbreak will face more direct threats than residents in lightly affected or non-affected regions (Zhang et al., 2020). High threat perceptions lead people to pay more attention to pandemic-related information and to process information more rigorously in terms of availability and depth (Sarkees et al., 2021). Therefore, people at the epicentre of COVID-19 generally hold higher risk perceptions of pandemic severity than those far from the epicentre and may be susceptible to a ripple effect (Liang et al., 2020). We postulate that COVID-19 severity will moderate the effect of a duty-free policy on travel intention as follows:

H2a: When consumers perceive low COVID-19 severity, tourist destinations with a duty-free policy will elicit stronger travel intentions than destinations without such a policy.
H2b: When consumers perceive high COVID-19 severity, tourist destinations with a duty-free policy will not elicit stronger travel intentions than destinations without such a policy.

### Moderating Role of Counterfactual Thinking

Coronavirus disease 2019is an unprecedented infectious disease that people are still seeking to understand (e.g., how is it transmitted? What types of isolation can prevent and control it? What treatment options are available?). The pandemic primarily hindered tourists’ travel decisions until self-quarantine or social distancing policies were publicised. Tourists were thus apt to engage in counterfactual thinking and evaluate their prior travel decisions (e.g., “If I had known about the COVID-19 outbreak earlier, I would not have scheduled my trip this summer.”).

The functional theory of counterfactual thinking (Epstude and Roese, 2008) indicates that such thinking can inform behavioural intention. Specifically, if people do not obtain an expected outcome, they can easily experience emotions such as regret. These reactions promote counterfactual thinking (i.e., envisioning how things could have turned out differently). Such thinking further inspires people to consider how they might achieve their anticipated outcome, sparking behavioural intentions to do so. For example, if a person has counterfactual thoughts about failing to travel when pandemic severity is relatively low, the person may hedge against the negative effects of COVID-19 on travel intentions and be driven to travel. However, if pandemic severity is relatively high, this promoting effect of counterfactual thinking on travel intention may decline or cease to exist. The following hypotheses are put forth accordingly:

H3a: For tourists with strong counterfactual thinking about the COVID-19 pandemic, destinations implementing a duty-free policy will generate higher perceived value and travel intentions than destinations without such a policy.
H3b: For tourists with weak counterfactual thinking about the COVID-19 pandemic, destinations with and without a duty-free policy will demonstrate no difference in promoting perceived value and travel intentions.

## Experiment

### Design and Materials

Four hundred and ten participants took part in this experiment, which involved a 2 (duty-free policy: absent vs. present) × 2 (COVID-19 severity: high vs. low) design. Participants were assigned to one of four experimental conditions. We recruited our participants from a popular online survey platform in China, Wenjuanxing; this site is similar to SurveyMonkey and Amazon Mechanical Turk. The samples for the study reported in this paper were recruited from Wenjuanxing’s more than one million members. Wenjuanxing has an equal proportion of male and female members who hail from all regions of China and have a variety of occupations. As a professional survey website, Wenjuanxing’s sample is valid, diverse, and representative.

Our sampling plan is random and convenient sampling through the online subject pool. The questionnaire was posted to the online subject pool. The data was collected from November 24, 2021 to December 25, 2021. Participants are recruited from both areas of high and low COVID-19 risk using the online subject pool. As for the definition of COVID-19 risk areas, we used the updated information on the Chinese Epidemic Control Center, as the basis for defining high-risk and low-risk areas. According to the definition, low-risk areas are defined as areas that have no new confirmed cases for 14 consecutive days; high-risk areas are defined as areas having more than 50 cumulative confirmed cases and an aggregated outbreak occurring within 14 days. During the data collection period, Liaoning province was the high-risk area of COVID-19 in China. On the other hand, the tourism destination elaborated in the experimental materials is Hainan Province, the only province in China that implements duty-free policy.

The independent variable (duty-free policy: absent vs. present) was manipulated by designing two reading materials about tourist destinations with or without a duty-free policy based on different conditions. The moderating variable (COVID-19 severity) was manipulated by recruiting participants who were or were not in a COVID-19 risk area. China’s Liaoning province was experiencing a COVID-19 outbreak at the time of this experiment. Participants from this province were therefore assigned to the high COVID-19 severity condition, while those from other provinces were assigned to the low COVID-19 severity condition. Another moderating variable (high or low counterfactual thinking about the pandemic) was measured by participants’ responses to a Likert scale rather than by experimental control. The mediating variable (consumers’ perceived value) was assessed by simulating participants’ purchase decisions as measured on an established scale. The dependent variable was evaluated by adapting a destination travel intention scale regarding tourists’ satisfaction, involvement, destination image, and revisit intentions.

### Procedures

First, all participants read a brief introduction to the COVID-19 pandemic and learned about the pandemic’s adverse effects. Participants also read about associated travel problems and about pandemic control measures. Next, each participant reported their level of counterfactual thinking about travel inconvenience due to the outbreak (e.g., “If the COVID-19 outbreak had not occurred in the past year, I could have enjoyed very free travel/shopping”).

Participants in the different experimental groups then read corresponding materials about a particular destination. Depending on the group, materials described a destination with or without a duty-free policy. All participants subsequently made simulated purchase decisions. Each participant was granted a travel shopping budget of 5,000 RMB and the freedom to choose from displayed options along with several featured products and duty-free items. Finally, participants reported their perceived transaction value regarding their simulated purchases and intentions to travel to the described destination. Table 1 lists all measurement items and their sources. Table 2 summarises the sample’s demographics, including participants’ age (*M*: 28 years), monthly income in RMB (*M*: 8149.64 yuan), gender (64.63% women, 35.37% men), and education level (mainly undergraduate).

**TABLE 1:** Measurement items and sources.

Constructs	Measurement items	Sources	
Counterfactual thinking	1. If the COVID-19 outbreak had not occurred in the past year, I could have enjoyed very free travel/shopping	Allen et al., 2014	α = 0.893
	2. If the COVID-19 outbreak had not been so severe in the past year, I could have enjoyed free travel/shopping		
	3. I would have been able to travel/shop freely without being restricted if COVID-19 controls had not been strict in the past year		
Consumers’ perceived value	4. I consider my travel purchases very practical	Spotts and Stynes, 1985; Lee and Crompton, 1992; Oppermann and Chon, 1997; Jang and Feng, 2007; Gallarza et al., 2013	α = 0.872
	5. I feel that the items I purchased during travel are fairly priced		
	6. I would have a similar purchase plan if I travelled again		
	7. To a large extent, I will share this travel experience with my friends and happily recommend my shopping experience		
Travel intention	8. The above tourist destination has elements that appeal to you 9. The above material inspires you to want to learn more about this tourist destination	Oh and Mount, 1998; Chen and Tsai, 2007; Chi and Qu, 2008; Kim et al., 2011	α = 0.869
	10. I did not know anything about this tourist destination beforehand, but after reading the description, I plan to make this place my next travel destination		
	11. After completing this questionnaire, I will share the information I just read with my friends as a destination for future travel		

**TABLE 2:** Sample demographics.

*N* = 410	Frequency (mean)	Percentage (standard deviation)
Age	28	5.42
Monthly income (RMB)	8149.64	8661.72
**Gender**		
Male	145	35.37
Female	265	64.63
**Education level**		
Less than bachelor’s degree	32	7.81
Bachelor’s degree	348	84.87
Master’s degree	27	6.59
Ph.D.	3	0.73

### Mediating Role of Consumers’ Perceived Value

To examine the potential mediating role of consumers’ perceived value, we carried out causal stepwise regression (Wen et al., 2022) and bootstrap sampling (Hesterberg, 2011) for mediation analysis. Results revealed a full mediating effect of consumers’ perceived value on the impact of a duty-free policy on travel intention. Causal stepwise regression and bootstrap sampling analyses are detailed in Tables 3, 4, respectively.

**TABLE 3:** Causal stepwise regression analysis of mediating effect.

	Travel intention	Consumers’ perceived value	Travel intention
Constants	5.586**	5.328**	2.552**
	−29.661	−28.829	−9.365
Duty-free policy	−0.267*	−0.346**	−0.07
	*t* = (−2.317)	*t* = (−3.060)	*t* = (−0.723)
Consumers’ perceived value			0.569**
			−13.594
*N*	410	410	410
*R* ^2^	0.013	0.022	0.321
Adjusted*R*^2^	0.011	0.02	0.318
*F*	*F*(1,408) = 5.369, *p* = 0.021	*F*(1,408) = 9.361, *p* = 0.002	*F*(2,407) = 96.286, *p* = 0.000

*p < 0.05.

**p < 0.01.

**TABLE 4:** Bootstrap sampling method.

c	a	b	a*b	a*b	a*b	a*b	a*b	c’	Conclusion
Total effect			Mediating effect value	Boot SE	*z* value	*p* value	95% BootCI	Direct effect	
−0.267*	−0.346**	0.569**	−0.197	0.001	−140.525	0	−0.140 to −0.028	−0.07	Full mediating effect

*p < 0.05 and **p < 0.01.

In sum, these findings suggest that a duty-free policy significantly increased participants’ intentions to travel to the specified destinations, with the effect being fully mediated by consumers’ perceived value. Next, we tested whether this mediating effect was moderated by (a) participants’ counterfactual thinking about COVID-19 and (b) whether a destination represented a COVID-19 risk area.

### Moderating Role of Counterfactual Thinking

We developed three models to analyse the moderating effect of counterfactual thinking. The independent variable (duty-free policy) was treated as a virtual variable, the moderating variable (counterfactual thinking) was centralised, and the dependent variable (consumers’ perceived value) remained unchanged. Model 1 considered the effect of a duty-free policy on consumers’ perceived value; Model 2 added the moderating variable to Model 1; and Model 3 contained the interaction term from Model 2 and integrated the moderating and independent variables. The *F* values changed significantly from Model 2 to Model 3, and the interaction term was significant as well; we thus observed a moderating effect of counterfactual thinking on the main effect. Results are outlined in Table 5.

**TABLE 5:** Analysis of counterfactual thinking moderating effect.

	Model 1	Model 2	Model 3
Constants	4.982**	4.921**	4.908**
	−59.046	−65.168	−64.987
Duty-free policy—1.0 [reference item]	–	–	–
Duty-free policy—2.0	−0.346**	−0.237*	−0.232*
	*t* = −3.060	*t* = −2.334	*t* = −2.295
Counterfactual thinking		0.383**	0.466**
		-10.27	-8.377
Duty-free policy—2.0* Counterfactual thinking			−0.150*
			*t* = −2.013
*N*	410	410	410
*R*^2^	0.022	0.224	0.231
Adjusted *R*^2^	0.02	0.22	0.226
*F*	*F*(1,408) = 9.361, *p* = 0.002	*F*(2,407) = 58.618, *p* = 0.000	*F*(3,406) = 40.722, *p* = 0.000
Δ*R* ^2^	0.022	0.201	0.008
Δ*F*	*F*(1,408) = 9.361, *p* = 0.002	*F*(1,407) = 105.479, *p* = 0.000	*F*(1,406) = 4.050, *p* = 0.045

* p < 0.05.

** p < 0.01.

### Moderating Role of COVID-19 Severity

The independent variable (presence or absence of a duty-free policy) and the moderating variable (COVID-19 risk area or not) were each binary variables. As such, we conducted a two-way analysis of variance to test the moderating effect of COVID-19 severity. The interaction term was significant, indicating a moderating effect. COVID-19 severity hence moderated the main effect; that is, a duty-free policy had less impact on travel intention under high COVID-19 severity than under low COVID-19 severity. This analysis is summarised in Table 6. Overall hypothesis testing results are displayed in Table 7.

**TABLE 6:** Analysis of COVID-19 moderating effect.

		*df*	*MS*	*F*	*p*
Correction Model	37.985a	3	12.662	10.224	0.000
Intercept	9123.723	1	9,123.723	7,367.078	0.000
COVID-19 severity × Duty-free policy	5.786	1	5.786	4.672	0.031
COVID-19 severity	22.245	1	22.245	17.962	0.000
Duty-free policy	10.357	1	10.357	8.363	0.004
Errors	502.809	406	1.238		
Total	9946.438	410			
Corrected Total	540.793	409			

*R^2^ = 0.070 (adjusted R^2^ = 0.063).*

**TABLE 7:** Hypothesis testing results.

Serial Number	Research hypothesis	Result
H1	Compared to the absence of a duty-free policy in a tourist destination, the implementation of a duty-free policy will generate stronger travel intention through greater perceived value	Accepted
H2a	When consumers perceive low COVID-19 severity, tourist destinations with a duty-free policy will elicit stronger travel intentions than destinations without such a policy	Accepted
H2b	When consumers perceive high COVID-19 severity, tourist destinations with a duty-free policy will not elicit stronger travel intention than destinations without such a policy	Accepted
H3a	For tourists with strong counterfactual thinking about the COVID-19 pandemic, destinations implementing a duty-free policy will generate higher perceived value and travel intentions than destinations without such a policy	Accepted
H3b	For tourists with weak counterfactual thinking about the COVID-19 pandemic, destinations with and without a duty-free policy will demonstrate no difference in promoting perceived value and travel intentions	Accepted

## General Discussion

### Conclusion

Consumer behaviour studies represent a key area of tourism research; travel intention is a pillar of consumer behaviour in this context. Inspired by earlier work, we explored factors influencing individuals’ travel intentions. The following conclusions can be drawn.

First, our experiments and data analysis demonstrated the impact and mechanism of a duty-free policy on individuals’ intentions to visit a tourist destination. Implementation of a duty-free policy in tourist destinations positively influenced travel intention: destinations with a duty-free policy inspired stronger travel intention than those without such a policy. Tourists’ perceived value also mediated the effect of a duty-free policy on travel intentions.

Second, COVID-19 severity and counterfactual thinking played moderating roles. When tourists faced low COVID-19 severity, destinations with a duty-free policy elicited stronger travel intentions than destinations without such a policy; when tourists encountered high COVID-19 severity, destinations with a duty-free policy did not elicit stronger travel intentions than destinations without this policy. Tourists with strong counterfactual thinking about the pandemic exhibited higher perceived value and travel intentions for destinations implementing a duty-free policy than for destinations without the policy. Tourists with low counterfactual thinking about the pandemic were indifferent (in terms of perceived value and travel intentions) about destinations that did and did not implement a duty-free policy.

### Theoretical Contributions

This research extends the functional theory perspective of counterfactual thinking, linking counterfactual thinking’s preparatory function for behavioural intention with tourists’ travel intentions. Our work thus introduces a fresh theoretical perspective on travel intention. Prior studies mainly investigated the facilitating effect of tourists’ perceived value on travel intentions vis-à-vis perceived value theory.

We have also expanded the application of perceived value theory. Few scholars have considered the impact of perceived value on tourists’ travel intentions amid the pandemic. We identified perceived value as an antecedent of travel intention, echoing previous research (e.g., Kozak, 2001; Lee S. Y. et al., 2007). Travel intention is not simply dependent on perceived material value; more importantly, such intention arises from affective values such as pleasure and aesthetics (Petrick, 2004; Gallarza and Gil Saura, 2006; Lee C. et al., 2007). Tourists tend to travel in pursuit of these values (Kirillova et al., 2014).

Discussions about promoting travel intention based on perceived value theory have typically ignored the facilitating effects of negative emotions on travel intention and instead focused on positive perceived affective values (e.g., pleasure and joy). However, the COVID-19 pandemic continues to evoke negative reactions (Carstensen et al., 2020; Lades et al., 2020). Our research bridges this gap by considering the content-neutral pathway of counterfactual thinking’s preparatory function for behavioural intention. Along this pathway, behavioural intention is derived from the mindset, emotions, and motivations resulting from counterfactual thinking (compared with behavioural intention arising from specific information in counterfactual thinking) which affect subsequent behaviour (Smallman and Roese, 2009; Roese and Epstude, 2017). Markman et al. (2008) found that the negative affect generated by counterfactual thinking can inspire people to change their behaviour: emotions such as sadness and regret (e.g., “I feel sad that I could have done better”; “I feel regret because I could have finished the task better”) can lead people to work harder on subsequent tasks. Our results suggest that travel intention may be sparked by negative emotions brought on by counterfactual thinking (e.g., imagining potential travel plans if the pandemic situation had been different). This pattern supports a content-neutral pathway.

In addition, the identified moderating role of counterfactual thinking substantiates the preparatory function of counterfactual thinking for behavioural intention. This moderation especially reinforces the content-specific pathway of counterfactual thinking’s preparatory function (Roese and Olson, 1997; Segura and Morris, 2005; Epstude and Roese, 2008). The pathway contains particular information for counterfactual thinking, thereby generating subsequent behavioural intention and spurring behaviour. “Content-specific” in this sense suggests that certain information directly affects behavioural intention: the more precise the intention obtained through counterfactual thinking, the more likely corresponding behaviour is to change. Counterfactual thinking research (e.g., Krishnamurthy and Sivaraman, 2002; Page and Colby, 2003) has demonstrated a positive role of such thinking on behavioural intention. Even so, it remains unclear whether this benefit is due to the content of counterfactual thinking (i.e., which influences behavioural intention) or to the negative emotions that stimulate behavioural intention (Morris and Moore, 2000; Sirois et al., 2010). Our experiments indicated that the specific content of counterfactual statements (i.e., about participants’ travel intentions amid the pandemic) shaped travel intention. This outcome further verifies the content-specific pathway of counterfactual thinking’s preparatory function.

Finally, our results confirm risk’s impact on travel intention and enrich the body of knowledge regarding how moderators of risk influence this intention. The adverse effect of risk on travel intention has been well documented: the higher a destination’s risk severity, the weaker tourists’ intentions to visit (e.g., Sheng-Hshiung et al., 1997; Fuchs and Reichel, 2006; Petrick et al., 2007). Our findings align with prior work in this respect. Conversely, research on moderators affecting travel intention has not addressed the moderating impact of counterfactual thinking arising from negative outcomes; such work has primarily discussed the moderating roles of positive psychological variables such as self-efficacy (e.g., Guo et al., 2016) and satisfaction (e.g., Yüksel and Yüksel, 2007). For example, Guo et al. (2016) found that tourists’ self-efficacy moderated the negative effect of tourists’ hostility on travel intentions. Our discovery—that counterfactual thinking (attributable to the COVID-19 pandemic) can positively moderate the negative impact of risk on travel intention—expands knowledge of the moderators of travel intention.

### Managerial Implications

Our findings provide a foundation for tourism companies to segment markets, select target markets, conduct market positioning, and implement marketing strategies under the background of COVID-19. Several actionable implications follow.

First, our results can guide tourism resource development. By examining the factors influencing tourists’ intentions to visit a destination, local governments and tourism companies responsible for developing tourism products can better understand affiliated locations. Comprehending tourists’ travel intentions can also help local governments and tourism companies to enhance tourism infrastructure and to create innovative products. Destinations’ tourism products can thus become more effective.

Second, this research reveals the direct effect of a duty-free policy on tourists’ travel intentions. Whereas a duty-free shopping policy may discourage visitation from tourists who prefer shopping-oriented trips, destinations can still attract potential tourists through such a policy. Specifically, if tourist destinations wish to further expand the impact of a duty-free policy, they should continue easing policy restrictions (including constraints on the number of duty-free shopping trips, duty-free shopping quotas, and the types and quantities of duty-free goods) while improving the duty-free shopping system to stimulate prospective tourists’ travel intentions. In addition, governments should plan to establish auxiliary facilities (e.g., cafes or leisure and entertainment sites) in duty-free stores’ surroundings; doing so can create a shopping system based on duty-free shopping.

Lastly, we considered the indirect effect of a duty-free policy on travel intention through perceived value. Findings suggest that tourist destinations should increase their perceived value *via* appropriate marketing tools. Once destination marketers understand tourists’ consumption needs based on perceived destination values, these personnel can develop and design tourism products tailored to target markets (e.g., by being market-oriented and implementing suitable marketing tools based on tourists’ value preferences). Destination marketers should also bear in mind that the COVID-19 pandemic is not over; associated risk perceptions can undermine destinations’ perceived value as well as tourists’ travel intentions. Tourism marketers should thus ponder ways to assuage tourists’ anxiety at different COVID-19 severity levels.

### Future Research Directions

Several limitations of our research leave room for future work. Subsequent studies could further categorise counterfactual thinking about the pandemic, such as by examining the relationships among different types of counterfactual thinking and travel intentions along with other relevant variables. Counterfactual thinking could specifically be divided into upward counterfactual thinking (i.e., envisioning how past outcomes could have been better) and downward counterfactual thinking (i.e., envisioning how past outcomes could have been worse) (Roese, 1994). Structurally, counterfactual thinking can be classified as either additive counterfactual (i.e., imagining how past outcomes could have been different if some antecedents were added) or subtractive (i.e., imagining how past outcomes could have been different if some antecedents were subtracted) (Roese et al., 1999). Prior research (e.g., Sirois et al., 2010) indicated that upward counterfactual thinking is more likely to promote behavioural intention than downward counterfactual thinking. This supposition can be further scrutinised.

Furthermore, we focussed on COVID-19 severity in China during a certain period. Cross-cultural studies could more fully delineate the impact of pandemic severity on travel intention. For example, researchers can build statistical models informed by COVID-19 big data to quantify pandemic severity in different regions. Additionally, our findings offer evidence of a ripple effect and a psychological typhoon eye effect, consistent with risk perception studies (e.g., Burns and Slovic, 2012; Wen et al., 2020). Scholars can further explore the mechanisms by which these two psychological effects occur in response to the same crisis event.

Lastly, in this paper, we only collected data from a popular online survey platform in China, Wenjuanxing. Due to that users who register for Wenjuanxing survey platform are mainly college students as well as young adults. We acknowledge that this composition of participants undermines the representativeness of our sample. For future research, we hope to remedy this deficiency by collecting a wider range of data or by including second-hand data from other sources (e.g., public data on the internet).

## Data Availability Statement

The original contributions presented in this study are included in the article/supplementary material, further inquiries can be directed to the corresponding author.

## Ethics Statement

Ethical review and approval was not required for the study on human participants in accordance with the local legislation and institutional requirements. The patients/participants provided their written informed consent to participate in this study.

## Author Contributions

YJX and YBX conceived to the main idea of the experiment, collected the data, conducted the data analyses, and wrote the main draft of this manuscript. WM and XX discussed the results and revised the manuscript together. All authors contributed to the article and approved the submitted version.

## Conflict of Interest

The authors declare that the research was conducted in the absence of any commercial or financial relationships that could be construed as a potential conflict of interest.

## Publisher’s Note

All claims expressed in this article are solely those of the authors and do not necessarily represent those of their affiliated organizations, or those of the publisher, the editors and the reviewers. Any product that may be evaluated in this article, or claim that may be made by its manufacturer, is not guaranteed or endorsed by the publisher.
